# Utility of Corrected Effective Atomic Numbers in Differentiating Hepatocellular Carcinomas and Liver Metastases From Hepatic Hemangiomas

**DOI:** 10.7759/cureus.82478

**Published:** 2025-04-17

**Authors:** Mitsunari Maruyama, Shota Hosogoshi, Minako Maruyama, Hisatoshi Araki, Rika Yoshida, Shinji Ando, Megumi Nakamura, Takeshi Yoshizako, Yasushi Kaji

**Affiliations:** 1 Department of Radiology, Shimane University Faculty of Medicine, Izumo, JPN

**Keywords:** corrected effective atomic number, dual-energy ct, hepatic hemagioma, hepatocellular carcinoma, liver metastasis

## Abstract

Objective

To examine whether malignant liver masses (hepatocellular carcinomas (HCCs)/liver metastases) can be differentiated from hepatic hemangiomas using corrected effective atomic numbers, which can eliminate the effect of fat on unenhanced CT images.

Materials and methods

An unenhanced and contrast-enhanced dual-energy CT scan was performed on 94 patients with 132 liver masses. Regions of interest with a diameter of 10 mm were placed in tumor areas without degeneration based on contrast-enhanced CT images. Corrected effective atomic numbers (Zeff) were calculated from the actual Zeff and liver fat value on unenhanced CT images using the following formula: corrected Zeff = [actual Zeff - 6.27 * liver fat value (%) / 100] / [1 - liver fat value (%) / 100]. The mean corrected Zeff of the HCC, liver metastasis, and hepatic hemangioma groups was compared.

Results

There were 43 HCCs, 40 metastases (17 from colon cancer and 23 from pancreatic cancer), and 49 hemangiomas. The mean corrected Zeff values were as follows: HCC 7.771 ± 0.123; metastasis 7.756 ± 0.204; and hemangioma 7.618 ± 0.114. A significant difference in corrected Zeff was found between hemangioma and both HCC and metastasis (p < 0.001), while no significant difference was observed between HCC and metastasis. Corrected Zeff≥7.666 distinguished malignant liver masses (HCC/metastasis) from hemangiomas with a sensitivity of 80.72% and specificity of 71.43% (AUC=0.771, 95% CI: 0.689-0.852).

Conclusions

A significant difference in corrected effective atomic number was found between malignant liver masses (hepatocellular carcinomas/liver metastases) and hepatic hemangiomas. No significant difference was found between hepatocellular carcinomas and liver metastases. A corrected Zeff ≥ 7.666 differentiated malignant liver masses from hepatic hemangiomas with 80.72% sensitivity and 71.43% specificity (AUC = 0.771).

## Introduction

Dual-energy CT scans can generate material-specific images that display the distribution and concentration of a specific material, such as iodine or fat [1,2]. It can also generate different monochromatic images to enhance soft-tissue contrast [3,4]. Material decomposition is a dual-energy CT analysis technique that decomposes dual-energy CT images into material-specific images. Material-decomposed images can provide increased contrast in visualizing arterial hyperenhancement and washout in hepatocellular carcinoma (HCC) [5,6]. Several studies have shown that dual-energy CT scans improved the early detection of hypervascular liver tumors [6-8].

Several studies have used dual-energy CT scans to differentiate liver masses. However, quantitative parameters such as normalized iodine concentration, which is used for differentiation, can be obtained using contrast-enhanced CT scans [9,10]. Hepatocellular carcinoma is the most common primary liver cancer. The incidence of liver metastasis is 18 to 40 times higher than that of primary liver cancer. Hepatic hemangioma is the most common type of benign hepatic lesion [10,11]. Distinguishing the above-mentioned liver masses using only unenhanced CT is challenging. Contrast-enhanced CT may not be feasible for patients with contrast material allergies or renal impairment. Further diagnostic examinations, such as ultrasound or MRI, may also not be feasible for patients who are unable to follow instructions. In such cases, the ability of unenhanced CT to determine whether an incidental liver mass is benign or malignant would be highly valuable.

There are no reports on the use of effective atomic numbers in differentiating liver masses using unenhanced CT scans alone. The measurement of effective atomic numbers by drawing regions of interest (ROIs) is convenient and easy. However, the fat component of liver tumors might affect the effective atomic number within the ROI. The effective atomic number of fat is low. Thus, the presence of fat lowers the effective atomic number within the ROI. A multimaterial decomposition (MMD) algorithm has been utilized to quantify liver fat in single-source rapid kilovoltage-switching dual-energy CT scans [12]. The Gemstone Spectral Imaging (GSI) (GE Healthcare, Chicago, IL, USA) liver fat algorithm (maximum mean discrepancy algorithm) can accurately perform liver fat quantification and is reproducible across scan phases [13].

We hypothesized that liver masses can be differentiated by calculating the corrected effective atomic number of liver masses, which eliminates the effect of fat via liver fat quantification. The current study aimed to examine whether malignant liver masses (hepatocellular carcinomas/liver metastases) can be differentiated from hepatic hemangiomas using corrected effective atomic numbers, which can eliminate the effect of fat on unenhanced CT scan images.

## Materials and methods

Patients

Multi-phase (unenhanced and three-phase contrast-enhanced) dual-energy CT scans were performed on 94 patients (132 liver masses) using a 256-row CT scan device (Revolution Apex Elite; GE HealthCare) from October 2023 to March 2024. This retrospective single-center study was approved by the Ethics Committee of Shimane University Faculty of Medicine, Izumo, JPN (approval no. 20240217-1). All participants provided informed consent.

Multi-phase dual-energy CT scan

Multi-phase dual-energy CT scan images were acquired. One unenhanced and three contrast-enhanced images were obtained. Table 1 shows the CT scan parameters. The patients received iodinated non-ionic contrast medium (600 mgI/kg) within 30 seconds using a power injector. The contrast material was iopamidol-370 (Iopamiron-370) or iomeprol-350 (Iomeron-350). Table 1 presents the amount and injection duration of the contrast material and scan duration.

**Table 1:** Scan parameters and scan protocol of the rapid kilovoltage-switching dual-energy CT scan GSI: Gemstone Spectral Imaging; TFI: TrueFidelity Imaging (GE HealthCare)

Parameters	Protocols
Scan parameters	
Tube voltage	80/140 kVp fast-switching
Tube current	GSI Assist (145-515 mA)
Noise index	11
Rotation time	0.5 second
Beam pitch	0.992: 1
Beam width	80 mm (detector coverage)
Image thickness	5.0 mm
Reconstruction algorithm	TFI-Medium
Reconstruction kernel	Standard
Contrast material	
Amount of contrast material	600 mgI/kg
Injection duration	30 seconds (via median cubital vein)
Scan phase	
Late arterial phase	12 seconds after the attenuation in the abdominal aorta reached 250 HU
Portal venous phase	30 seconds after late arterial phase
Equilibrium phase	110 seconds after portal venous phase

Three types of images were reconstructed from the unenhanced CT scan, which was a component of the multi-phase CT acquisition: 70-keV monochromatic images, effective atomic number (Zeff) images, and liver fat quantification images. The GSI liver fat algorithm for liver fat quantification uses the MMD algorithm with blood, fat, and liver tissue as materials [12,13]. The 70-keV monochromatic images were reconstructed from the contrast-enhanced CT scan acquisition.

Diagnostic procedures

The lesions were diagnosed based on the following common multi-phase CT scan findings: (1) HCC, presence of non-rim arterial phase hyperenhancement, non-peripheral washout, and enhanced capsule [14]; (2) metastasis, presence of hypovascular patterns, peripheral ring-like enhancement, and central necrosis [15]; (3) hemangioma, well-circumscribed lesions with peripheral nodular enhancement and progressive centripetal fill-in [16]. Finally, two observers with 14 and 16 years of experience in abdominal radiology characterized each lesion type as HCC, metastasis, and hemangioma.

Assessment

The region of interest (ROI) was placed in the lesions by a single radiologist who has 16 years of experience in abdominal radiology. The software (AW Server 3.2 Ext. 4.9; GE HealthCare) was used to establish and measure ROIs with a diameter of 10 mm in the tumor on 70-keV monochromatic unenhanced CT scan images. The ROI was placed in the axial section of the maximum diameter of the tumor, as close to the center of the tumor as possible. Tumors ≥10 mm were evaluated. The ROI was set in the area where there was no necrosis, referring to contrast-enhanced CT scan images that were consensually assessed by two observers with 14 and 16 years of experience in abdominal radiology.

The liver fat value indicates the percentage of fat within the ROI. In the GSI liver fat algorithm, the effective atomic number (Zeff) of fat (adipose tissue 3) was 6.27 [17]. The Zeff value within the ROI is calculated as the average value within the ROI. In addition, assuming that the ROI is composed of tumor parenchyma and fat, we used the percentage of fat to calculate corrected Zeff within the ROI, which removes the influence of fat. The corrected Zeff was calculated from the actual Zeff and liver fat value (%) on unenhanced CT scan images using the following formula: Actual Zeff-6.27*liver fat value (%)/100/1-liver fat value (%)/100

Statistical analysis

Data with normal distributions were expressed as means ± standard deviations, and those with non-normal distributions as medians (min-max). The Shapiro-Wilk test was used to assess the normality of data distribution. The mean corrected Zeff of the HCC, metastasis, and hemangioma groups was compared. The Tukey’s multiple comparison test was used to compare the mean corrected Zeff among the groups. The diagnostic capability of the mean corrected Zeff to distinguish malignant liver masses (HCC/metastasis) from hemangiomas was evaluated by calculating the area under the receiver operating characteristic curve (AUC). The Youden index was used to establish the optimal cut-off value for distinguishing between these two types of liver masses. A p-value of < 0.05 indicated statistically significant differences. All statistical analyses were performed using GraphPad Prism 9 (GraphPad Software Inc., San Diego, CA, USA).

## Results

**Figure 1:**
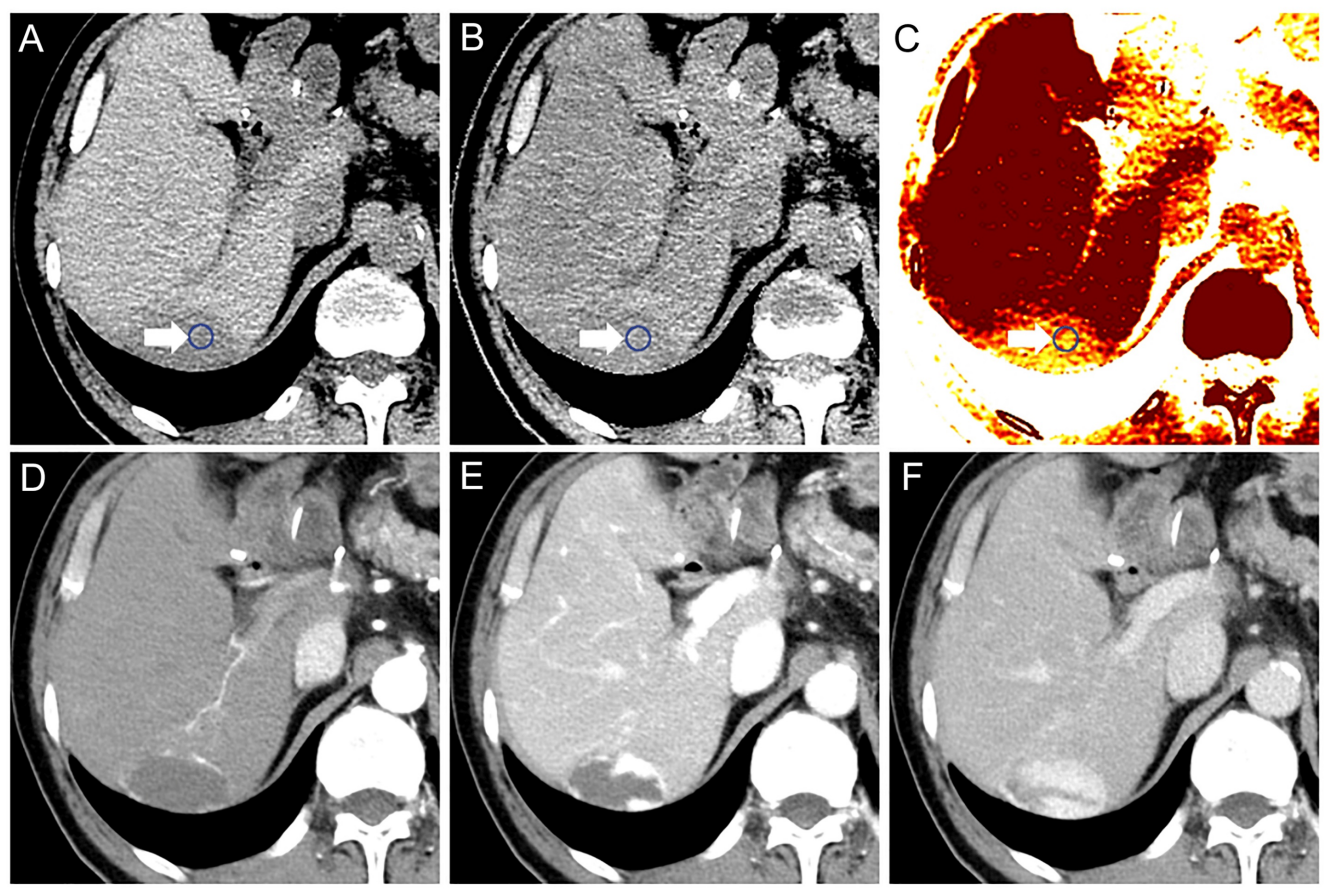
Representative case A: Unenhanced CT scan image; B: Zeff image; C:  Liver fat image; D: CE CT scan image late arterial phase; E: CE CT scan image portal venous phase; F: CE CT scan image equilibrium phase The lesion was characterized as a hepatic hemangioma. The corrected Zeff was calculated to eliminate the effect of fat. The white arrows point at the ROI, which was set in the area with no necrosis. The corrected Zeff was 7.622. CE: Contrast-enhanced, ROI: Region of interest

In total, 94 patients (73 men and 21 women) were included in the current analysis. The median age of the patients was 73 (41 to 96) years. Thirty-one patients presented with 43 HCCs, with a median size of 26.0 (12-176) mm. Further, 22 patients with 40 metastases with a median size of 21.5 (12-145) mm were included in the analysis. Of the 40 metastases, 17 were colon cancer metastatic lesions and 23 were pancreatic cancer metastatic lesions. Finally, 41 patients with 49 hemangiomas with a median size of 16.0 (12-76) mm were included in the analysis. The targeted patients and tumor numbers are listed in Table 2. The mean corrected Zeff of HCC, metastasis, and hemangioma were 7.771 ± 0.123, 7.756 ± 0.204, and 7.618 ± 0.114, respectively. Figure 1 shows the representative case.

**Table 2:** Targeted patients and number of tumors HCC: Hepatocellular carcinoma

Parameters	HCC	Metastasis (of the liver)	Hemangioma (hepatic)
Patients number	31	22	41
Tumor number	43	40	49
Tumor size (mm)	26.0 (12–176)	21.5 (12–145)	16.0 (12–76)

**Figure 2:**
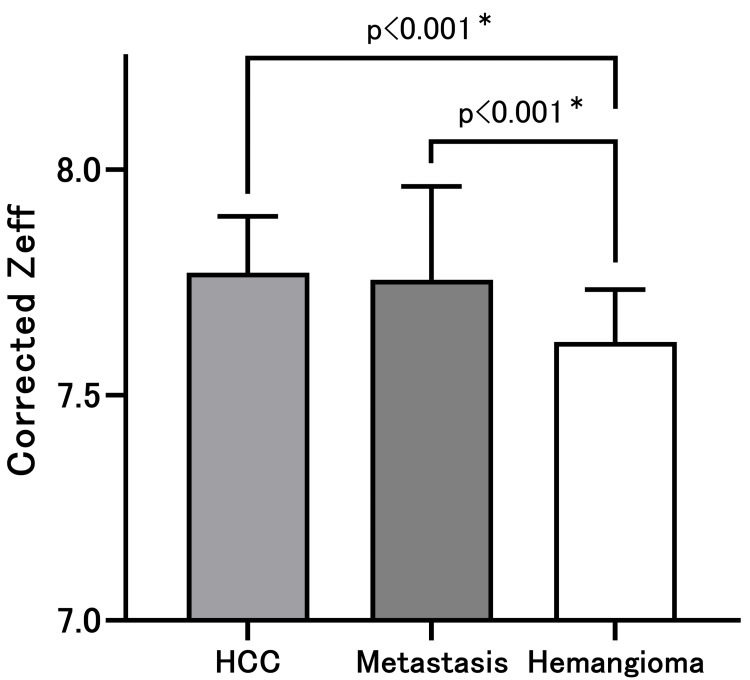
Corrected effective atomic numbers There was a significant difference in the corrected Zeff between hepatic hemangioma as well as HCC and metastasis of the liver. The corrected Zeff between HCC and liver metastasis did not significantly differ. HCC: Hepatocellular carcinoma; * Tukey‘s multiple comparison test

**Figure 3:**
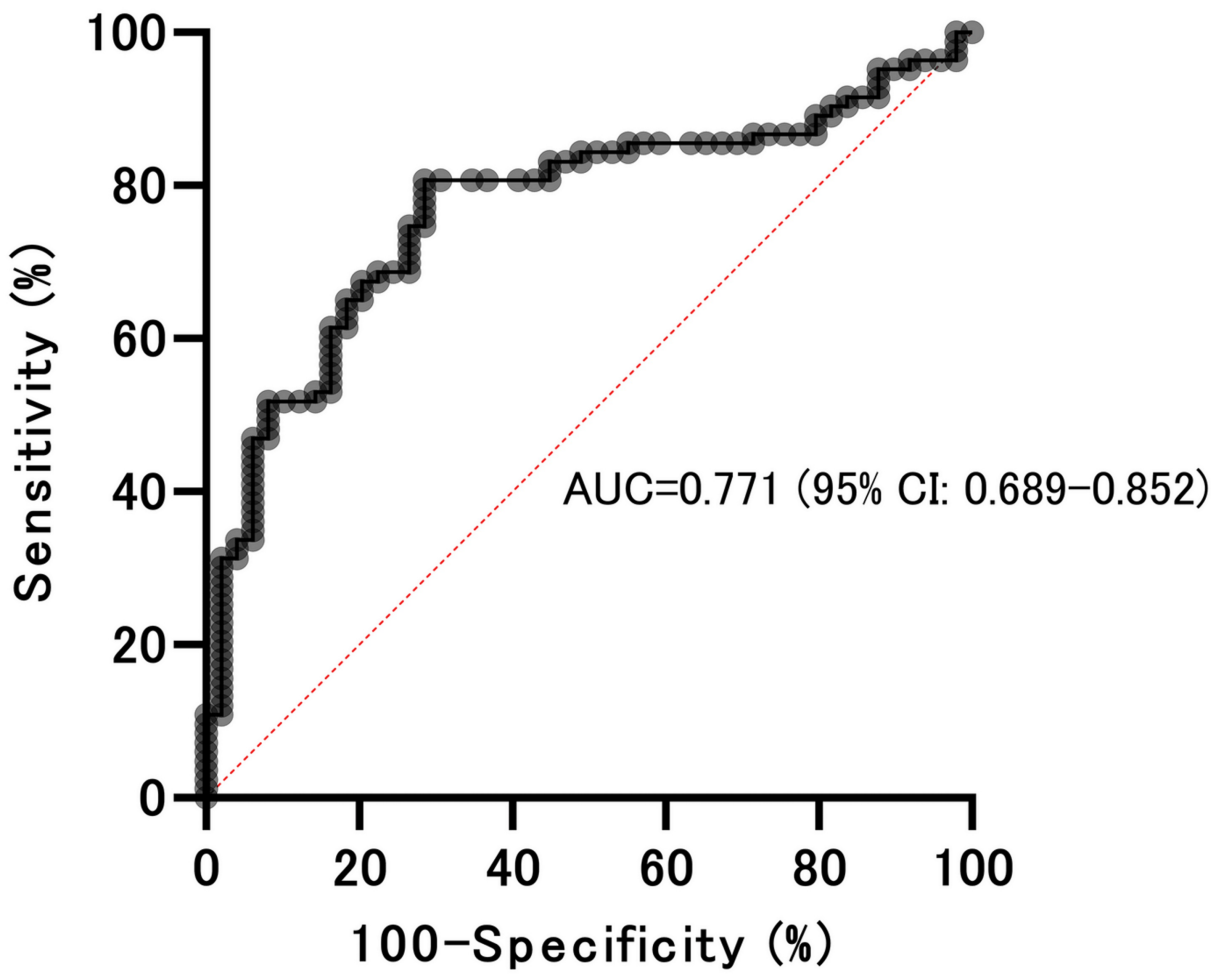
Receiver operating characteristic curves The diagnostic capability of the mean corrected Zeff to distinguish malignant liver masses (HCC/liver metastasis) from hepatic hemangiomas was evaluated by calculating the AUC. The Youden index was used to establish the optimal cut-off value for distinguishing between these two types of liver masses. A corrected Zeff ≥ 7.666 on unenhanced CT scan could distinguish malignant liver masses from hepatic hemangiomas, with a sensitivity of 80.72% (95% CI: 70.96-87.77%) and a specificity of 71.43% (95% CI: 57.59-82.15%). The AUC was 0.771 (95% CI: 0.689-0.852). HCC: Hepatocellular carcinoma; AUC: Area under the receiver operating characteristic curve; CI: Confidence interval

There was a significant difference in the corrected Zeff between HCC and hemangioma (p < 0.001) and between metastasis and hemangioma (p < 0.001); the Tukey’s multiple comparison test (Figure 2). The corrected Zeff between HCC and metastasis did not significantly differ. If the malignant liver masses were considered as HCC/metastasis, a corrected Zeff≥7.666 could differentiate malignant liver masses from hemangiomas, with a sensitivity of 80.72% (95% confidence interval (CI):70.96-87.77%) and a specificity of 71.43% (95% CI: 57.59-82.15%). The AUC was 0.771 (95% CI: 0.689-0.852) (Figure 3).

## Discussion

The current study showed that malignant liver masses (HCC/metastasis) could be differentiated from hemangiomas by calculating the corrected effective atomic number within the ROI, which is a simple and useful method. Several studies on the clinical application of effective atomic number imaging using GSI have been published. These include reports on the differentiation of benign from malignant lung masses [18], differentiation of renal cysts from renal tumors [19], differentiation of adrenal adenomas from adrenal metastases [20], assessment of sentinel limbus node metastasis in breast cancer [21], and differentiation of soft plaques from fibrotic plaques in the coronary arteries [22]. Based on most reports, effective atomic number imaging alone is not useful. However, it was used as an index in multiparametric analysis.

A significant difference in corrected effective atomic number was found between malignant liver masses and hepatic hemangioma. No significant difference was found between hepatocellular carcinomas and liver metastases, or between colon cancer metastases and pancreatic cancer metastases. The origin of the difference in the corrected effective atomic number between malignant liver masses and hepatic hemangioma might be influenced by pathological tissues. Hepatic hemangioma shows vascular proliferation, with blood filling the intravascular space and fibrous interstitium between the blood vessels. On the other hand, corrected effective atomic numbers of malignant liver masses might reflect tumor parenchyma. A corrected Zeff ≥ 7.666 differentiated malignant liver masses from hepatic hemangiomas with 80.72% sensitivity and 71.43% specificity (AUC = 0.771). Quantitative analysis of the tumor by volume of interest (VOI) and using spectral or photon-counting CT could provide deeper insight and is expected to improve the diagnostic performance of the corrected Zeff cut-off value. To further improve the diagnostic performance of the corrected Zeff cut-off value, it might be important to consider patient backgrounds, such as whether the patients have liver cirrhosis, nonalcoholic steatohepatitis, or cancers.

Liver masses such as HCC and metastasis contain fat. Several studies based on proton magnetic resonance spectroscopy have reported that most cancer cells contain mobile lipids. Tumor necrosis, apoptosis, hypoxia, and a combination of these mechanisms are the possible processes by which mobile lipids appear in the tumor microenvironment [23]. It might be predicted that the degree of fatty deposits in tumors would also differ. The liver fat value (%) in tumors ranged from 0 to 32 in the present study (HCC 8.54 (0.16-30.34); metastasis12.84 (2.05-32); hemangioma 5.09 (0-27.07)). Additionaly, the liver fat value (%) in metastasis was significantly higher than in hemangioma (p<0.001) and HCC (p=0.002, the Kruskal-Wallis test). There was no significant difference between the liver fat value (%) in hemangioma and HCC.

In the GSI liver fat algorithm, the effective atomic number of fat (adipose tissue 3) was 6.27 [17]. The effective atomic number of liver masses can be influenced by the fat content within the ROI. The corrected effective atomic numbers were calculated by subtracting the effect of fat. The GSI liver fat algorithm for liver fat quantification was accurate in the liver parenchyma. This algorithm is a material-based measurement of blood, fat, and liver tissue [12,13]. By contrast, the HCC fat fraction measured using this algorithm had weak to moderate correlations with the HCC fat fraction measured by chemical-shift MRI [24]. Tumors can have a different composition than the normal liver parenchyma. The influence of fat can be somewhat eliminated. However, it is challenging to accurately predict intratumoral fat content using the GSI liver fat algorithm. Metal deposition and internal microdegeneration in tumors might reduce the accuracy of predicting intratumoral fat. New algorithms considering metal deposition and microinternal degeneration, spectral or photon-counting CT, are expected to improve accuracy.

The current study had several limitations. First, the present study included a small number of patients and focused on only one type of benign liver mass (hemangioma), comparing it to two types of malignant liver masses (HCC, metastases from colon and pancreatic cancers). Second, the above group classification was based on multi-phase CT scan findings, without pathological evaluations. Third, the GSI liver fat algorithm might not be perfectly accurate for fat quantification in liver tumors. Quantitative analysis of the tumor regarding VOI and using spectral or photon-counting CT could provide deeper insight and is expected to improve accuracy. Finally, we did not evaluate radiologists' diagnostic performance with or without the use of the corrected Zeff cut-off value and therefore could not demonstrate its clinical usefulness in diagnosing liver tumors. Thus, further research must be performed.

## Conclusions

The corrected effective atomic number of malignant liver masses (hepatocellular carcinomas/liver metastases) significantly differed from hepatic hemangioma. There was no significant difference between hepatocellular carcinomas and liver metastases. A corrected Zeff ≥ 7.666 differentiated malignant liver masses from hepatic hemangiomas with good sensitivity and specificity.
